# MicroRNA-298 reduces levels of human amyloid-β precursor protein (APP), β-site APP-converting enzyme 1 (BACE1) and specific tau protein moieties

**DOI:** 10.1038/s41380-019-0610-2

**Published:** 2020-01-15

**Authors:** Nipun Chopra, Ruizhi Wang, Bryan Maloney, Kwangsik Nho, John S. Beck, Naemeh Pourshafie, Alexander Niculescu, Andrew J. Saykin, Carlo Rinaldi, Scott E. Counts, Debomoy K. Lahiri

**Affiliations:** 1 Laboratory of Molecular Neurogenetics, Department of Psychiatry, Indiana University School of Medicine, Indianapolis, IN USA; 2 Indiana Alzheimers Disease Center, Indiana University School of Medicine, Indianapolis, IN USA; 3 Departments of Radiology & Imaging Sciences, Indiana University School of Medicine, Indianapolis, IN USA; 4 Departments of Translational Neuroscience and Family Medicine, Michigan State University, Grand Rapids, MI USA; 5 Neurogenetics Branch, National Institute of Neurological Disorders and Stroke, National Institutes of Health, Bethesda, MD USA; 6 Stark Neuroscience Research Institute, Indiana University School of Medicine, Indianapolis, IN USA; 7 Department of Paediatrics, University of Oxford, South Parks Road, Oxford, OX1 3QX UK

**Keywords:** Neuroscience, Diseases

## Abstract

Alzheimer’s disease (AD) is the most common age-related form of dementia, associated with deposition of intracellular neuronal tangles consisting primarily of hyperphosphorylated microtubule-associated protein tau (p-tau) and extracellular plaques primarily comprising amyloid- β (Aβ) peptide. The p-tau tangle unit is a posttranslational modification of normal tau protein. Aβ is a neurotoxic peptide excised from the amyloid-β precursor protein (APP) by β-site APP-cleaving enzyme 1 (BACE1) and the γ-secretase complex. MicroRNAs (miRNAs) are short, single-stranded RNAs that modulate protein expression as part of the RNA-induced silencing complex (RISC). We identified miR-298 as a repressor of APP, BACE1, and the two primary forms of Aβ (Aβ40 and Aβ42) in a primary human cell culture model. Further, we discovered a novel effect of miR-298 on posttranslational levels of two specific tau moieties. Notably, miR-298 significantly reduced levels of ~55 and 50 kDa forms of the tau protein without significant alterations of total tau or other forms. In vivo overexpression of human miR-298 resulted in nonsignificant reduction of APP, BACE1, and tau in mice. Moreover, we identified two miR-298 SNPs associated with higher cerebrospinal fluid (CSF) p-tau and lower CSF Aβ42 levels in a cohort of human AD patients. Finally, levels of miR-298 varied in postmortem human temporal lobe between AD patients and age-matched non-AD controls. Our results suggest that miR-298 may be a suitable target for AD therapy.

## Introduction

Alzheimer’s disease (AD) is a complex neurodegenerative disorder with a phenotypic spectrum that includes memory loss as well as decline in other cognitive domains (e.g., executive function, language, perceptual-motor), functional decline and especially in later stages neuropsychiatric symptoms (e.g., irritability, depression, agitation, and hallucinations) [1]. Given that the strongest risk factor is age and the population of aging adults is increasing, the incidence of this disorder is expected to likewise rise dramatically. In 2019, an estimated 44 million cases of AD have been reported worldwide [2], with 5.8 million cases are estimated for within the USA, alone. It is estimated there will be one half to one million new cases within 2019, although at least half of those who already have AD will likely die due to the disorder within the same year, depending on estimation methods [3]. The neuropathology of AD is characterized by deposition of senile plaque and neurofibrillary tangles [4]. Senile plaques are generated by oligomerization of soluble β-amyloid (Aβ) peptide, which is generated by the sequential cleavage of amyloid- β precursor protein (APP) by β-site APP-cleaving enzyme 1 (BACE1) and the γ-secretase complex [5]. The most common forms of Aβ are 40 and 42 amino acid peptides, Aβ40 and Aβ42. Thus, reduction of soluble Aβ by targeting APP and BACE1 is a therapeutic goal, in accordance with the amyloid hypothesis [6]. However, both APP and BACE1 play an important role in normal physiological function of neuronal and glial cells [7–9]. APP acts in pancreatic islets [10], and BACE1 plays a role in normal liver metabolism [11–13]; therefore, a complete knockdown of either protein is undesirable. Moreover, attempts at inhibiting BACE1 or buffering Aβ directly have had limited success [11, 14] though work continues to identify selective inhibitors of upstream modulators of BACE1 expression. Therefore, a novel mechanism to reduce Aβ levels by modulating APP and/or BACE1 is warranted. An additional hallmark of AD is accumulation of hyperphosphorylated microtubule-associated protein tau [15] (gene: MAPT), (p-tau) in intraneuronal tangles. Any endogenous target that could potentially regulate both would be a valuable route of study for effective treatment.

Modulation of protein expression can be achieved by microRNAs (miRNAs), which often target the 3ʹ untranslated regions (UTR) of specific mRNAs and modify their expression post transcriptionally [16]. Specific miRNAs are differentially expressed in brain tissue from AD vs non-AD individuals [17–22]. Further, miRNAs also exist in biofluids such as urine, cerebrospinal fluid (CSF), blood and blood derivatives such as serum and plasma [19, 23, 24] from AD patients, thereby confirming a possible role as biomarkers for AD diagnosis.

miRNAs that modulate APP, BACE1, and tau have been identified, and the APP and BACE1-regulating miRNAs likewise reduce levels of soluble Aβ40. APP is regulated by miR-16 [25–27], miR-101 [28, 29], miR-153 [30, 31], miR-193b [32], and miR-200b [33]. Similarly, miR-9 [21], miR-15b [34, 35], miR-29a/b [21], miR-135a [33], miR-135b [36], miR-195 [37], and miR-339-5p [38] modulate BACE1 expression in different models. Tau is regulated by, among others, miR-16, miR-24c-5p, and miR-132 [39, 40]. To our knowledge, only miR-384 has been reported to target both APP and BACE1 [41] and miR-16 to target both APP and tau [25, 26, 40]. No miRNA has been reported, thus far, to regulate all three of these AD-associated proteins.

**Fig. 1:**
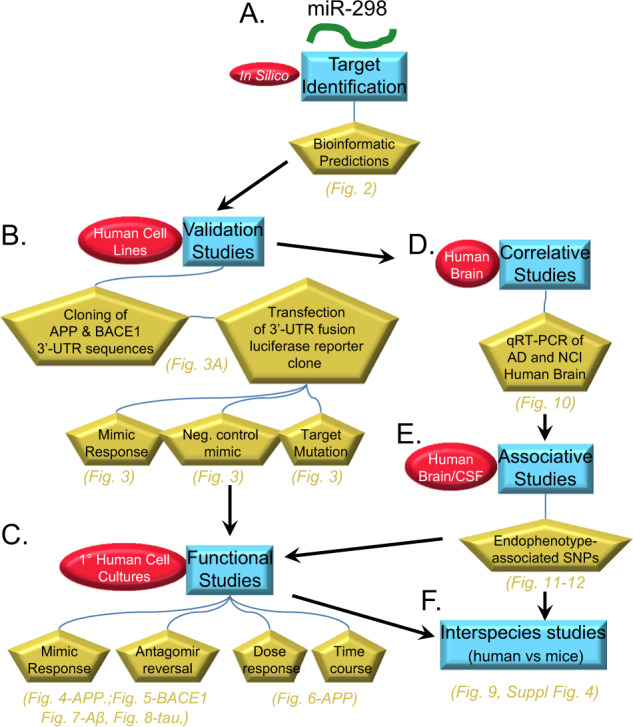
Overview of study design. This describes a comprehensive and integrated strategy of in silico identification, bioinformatics prediction and followed by the validation, functional, correlation, association and interspecies studies. In short, we report convergent supporting evidence for the proposed miR-298 effect, from human cell culture transfection and protein studies, human genetics, human brains, and animal model CNS. We have made a convincing case that this molecule deserves more attention and study as shown in subsequent figures. **a** miR-298 was identified as a potential candidate from rodent cell studies [42]. We used in silico database and prediction utilities to identify candidate target sites on the APP and BACE1 3ʹ-UTR sequences. These bioinformatic predictions informed further exploration. **b** We first created 3ʹ-UTR APP and BACE1 fusions in dual-reporter luciferase vectors, which were then optimized and used for validation studies. We co-transfected the clones into human-origin cell lines with miR-298 mimics, with miR-298 mimics plus antagomir inhibition, and mutated clones with miR-298 mimics. **c** We followed validation with functional studies, and measured levels of APP, BACE1, and tau proteins in cell lines and primary human brain cell cultures. We treated cultures with miR-298 mimics or mimics plus antagomirs to measure potential changes in target protein levels. **d** Upon validation of binding sites, we also tested levels of miR-298 in human control and AD brain samples, specifically by qRT-PCR. Surveys of APP and BACE1 protein levels in a portion of these brain samples have been published elsewhere [28, 38]. Investigation between miR-298-associated SNPs and endophenotypes of AD patients revealed that potentially important connections between miR-298-associated SNPS vs. Aβ levels and tau phosphorylation. This information led us to expand functional studies to include potential effects of miR-298 treatment on tau levels as well as APP and BACE1. **f** Finally, we compared interspecies responses in mouse models with mmu-miR-298.

miR-298, encoded by the *MIR298* gene, is a negative regulator of BACE1 in murine cells [42]. However, potential regulation of miR-298 on human BACE1 protein was not evaluated in that study. We, therefore, studied the effect of miR-298 on BACE1 expression in human cells. We found that miR-298 negatively regulates both APP and BACE1 expression, resulting in a reduction of soluble Aβ levels. To our surprise, we found miR-298 modulates expression of two specific tau isoforms, reiterating the potentially important role the miRNA may have in AD pathology. In vivo overexpression of human miR-298 by AAV-miR-298 construct resulted in nonsignificant reduction of APP, BACE1, and tau in mice. Finally, we measured levels of miR-298 in human brain (Brodmann areas—BA 21/22, aka middle and superior temporal gyri), cerebellum, and posterior cingulate cortex (PCC) from autopsied brains of both AD patients and age-matched non-AD controls. Notably, miR-298 levels vary in AD only in BA 21/22 samples. In summary, we report convergent supporting evidence for the proposed miR-298 effect, from human primary cell culture, human genetics, human brains, and animal model spinal cords (Fig. 1). We  posit that miR-298 deserves more attention and study.

## Materials and Methods

### Identification of putative miR-298 binding sites on the APP, BACE1, and MAPT 3′-UTR sequences

Multiple miRNA prediction utilities and databases [43–48] were consulted to probe the 3′-UTR sequences of APP and BACE1. Each used different algorithms to make determinations [49]. In addition, the RNAhybrid [50] utility was used to search for noncanonical miR-298 affinities with the tau 3′-UTR. To further characterize the discovered sites, we extracted selected mammalian sequence alignments from the UCSC Multiz alignment of 100 vertebrate genomes [51, 52].

### Cell culture

Human glioblastoma-astrocytoma (U373) and HeLa cells (obtained from ATCC, Manassas, VA) were grown in Minimum essential medium (MEM) containing 10% fetal bovine serum. Cells were trypsinized and counted by trypan-blue exclusion when ~70% confluent. A total of 150,000 cells/well and 50,000 cells/well were seeded onto 24-well and 96-well plates, respectively, for experiments. This sample size has been suitable for our previous work with miRNA [30, 53, 54].

### Human primary mixed-brain cultures

Human primary mixed-cell type brain cultures were grown in our lab by methods we previously developed [30, 55]. The fetal tissues were obtained from the Birth Defects Research Laboratory (BDRL) at the University of Washington with approval from the Indiana University school of Medicine Institutional Review Board (IRB). Briefly, brain tissues were cleaned of visible blood vessels and sectioned. Sections were digested with trypsin and gently triturated 10–12 times then centrifuged. Cells were resuspended and plated onto PDL-coated plates/wells, using supplemented neurobasal medium with antibiotics. Cells were propagated 20 DIV and transfected with RNAiMAX and Opti-MEM (ThermoFisher) as described previously [49]. This sample size has been suitable for our previous work with miRNA [30, 53, 54].

### Generation of APP 3′-UTR mutants

Target sites of miR-298 on the APP 3′-UTR were identified by Targetscan [43]. Each site was independently mutated using the QuikChange Lightning kit (Agilent). Briefly, primers were generated with regions overlapping the seven nucleotide target sites. The APP 3′-UTR [28] was denatured and mutagenic primers were extended with kit reagents. Parental DNA was digested by *Dpn I* and transformed into competent *Escherichia coli*. To confirm mutagenesis, plasmids were analyzed on a DNA gel, confirming the introduction of a novel enzyme site that replaced the miR-298 site. We did not successfully introduce mutation into the full-length BACE1 3′-UTR-containing reporter.

### DNA transfection and reporter assay

One day before transfection, about 50,000 cells/well of HeLa cells were seeded onto 96-well plate. A total of 300 ng of dual-luciferase psiCHECK 2 (Promega) plasmid containing full-length APP 3′-UTR [28], mutagenized APP 3′-UTR, or BACE1 3′-UTR [38] was independently co-transfected with 50 nM miR-298 using TransFectin (BioRad). Cells were incubated 48 h before being lysed for reporter assay. For mutagenesis experiments, 300 ng of the APP 3′-UTR containing the mutagenized site was co-transfected with miR-298.

### Transfection in 24-well plates

For U373 transfections, ~150,000 cells/well were seeded onto a 24-well plate. The following day, transfection complexes were made with RNAiMax (ThermoFisher) and 50 nM miRNA mimic or 20 nM siRNA in Opti-MEM media (ThermoFisher). Complexes were allowed to form for 20 min before being added to cells. Then 400 µl of media (no antibiotics) was added to each well. Mock-transfection consisted of Opti-Mem media as well as RNAiMax, but without miRNA mimics. Negative control mimic (NCM), from ThermoFisher was transfected at same nM concentration as test miRNA. Cells were incubated 72 h until lysis. For mixed primary cell transfection, 150,000 cells/well were plated and transfections were performed at DIV 17 using 75 nM miRNA mimic, 75 nM miRNA antagomir, and 50 nM siRNA. Cells were harvested 72 h post transfection unless described otherwise.

### Cell lysis and procurement of lysates

Cells were lysed using 100 µl mammalian protein extraction reagent (M-PER, Thermo) buffer containing protease inhibitor cocktail (Roche) and 10% sodium dodecyl sulfate (SDS). Cells were collected in 1.5 ml tubes and centrifuged at 11,000 *g* for 10 min to obtain supernatants. The supernatants were boiled using Laemmli sample buffer (LSB; final concentrations in lysate: 30 mM Tris, pH 6.8, 1% SDS, 12.5% glycerol, 0.005% bromophenol blue, 10% β-mercaptoethanol).

### SDS–polyacrylamide gel electrophoresis (SDS–PAGE) and western blotting

Equal amounts of denatured protein cell lysates were loaded onto 26-lane bis-tris criterion gels (BioRad) and separated using XT-Mops (BioRad) at 200V for ~70 min. Proteins were transferred onto a PVDF membrane using the iBlot system (ThermoFisher) and blocked using 5% milk in tris-buffered saline containing Tween-20 (TBST). Anti-APP (22C11, Millipore, 1:1000), anti-BACE1 (D10E5, Cell Signaling Technologies, 1:500) or anti-BACE1 (Abcam, 1:500), anti-Total tau (tau-5, ThermoFisher, 1:1000), anti-β-actin (Sigma, 1:500,000), anti-α-tubulin (Sigma, 1:500,000) were diluted in blocking agent (5% in milk/TBST) and probed separately overnight at 4 °C. After washing, appropriate horseradish peroxidase-conjugated secondary antibody was applied for 1 h and chemiluminescence (Pierce) was performed to obtain western blotting images. In order to study the effects of miR-298 on soluble APP (sAPP), conditioned media were analyzed on a gel and sAPP detected using 22C11.

### Assay of soluble Aβ40 and Aβ42 peptides by enzyme labeled immunosorbent assay (ELISA)

Using sandwich-based ELISA kits for Aβ40 and Aβ42 from IBL-America (Minneapolis, MN), levels of both soluble peptides were measured in the conditioned media of transfected cells. A standard curve was generated in accordance with the protocol to obtain pg/ml concentration of each well for each peptide.

### Human brain samples from AD and age-matched noncognitively impaired (NCI) controls

Age-matched samples of temporal lobe (TL) (BA 21/22) and cerebellum from control (CTL) and AD subjects representing both sexes were obtained from the University of Kentucky AD Research Center Brain Bank, whereas PCC (BA 23) was obtained from the Rush AD Research Center Brain Bank (Table 1). Exclusion criteria for cases selection at both sites included evidence of synucleinopathies such as Parkinson’s disease and Lewy body disease, frontotemporal dementia, argyrophilic grain disease, vascular dementia, hippocampal sclerosis, and/or large strokes or lacunes.

**Table 1:** Characteristics of human subjects

Subject	Source^a^	Age (yrs)	Sex	PMI^b^	Diagnosis^c^	MMSE^d^	Braak	Subject	Source	Age (yrs)	Sex	PMI	Diagnosis	MMSE	Braak
1	U KY	79	Female	3 3/4	AD	5	6	26	Rush	79	Male	7	NCI	30	1
2	U KY	69	Male	4	AD	4.5	6	27	Rush	92	Male	7 3/4	NCI	25	2
3	U KY	88	Male	3 3/4	AD	24	5	28	Rush	87	Male	6 3/4	NCI	29	4
4	U KY	86	Female	4	AD	14	6	29	Rush	91	Male	2 1/4	AD	24	4
5	U KY	83	Female	3 2/4	AD	5	6	30	Rush	82	Male	6	NCI	28	3
6	U KY	74	Male	3	AD	NA	6	31	Rush	86	Male	6 1/4	NCI	27	4
7	U KY	82	Female	4 3/4	AD	13.5	6	32	Rush	78	Male	9 3/4	NCI	29	2
8	U KY	65	Male	4	AD	0	6	33	Rush	94	Male	7 2/4	NCI	30	3
9	U KY	84	Female	5	NCI	30	3	34	Rush	93	Female	3 1/4	NCI	27	4
10	U KY	86	Male	3 1/4	AD	9.5	6	35	Rush	95	Female	8	AD	17	4
11	U KY	80	Female	3 3/4	AD	16	6	36	Rush	91	Female	3 3/4	AD	10	5
12	U KY	96	Male	3 2/4	AD	22	5	37	Rush	88	Female	3	AD	16	5
13	U KY	92	Female	3 2/4	AD	19	5	38	Rush	82	Female	8	NCI	27	3
14	U KY	76	Male	4 1/4	AD	27	6	39	Rush	95	Female	5 1/4	AD	20	5
15	U KY	79	Female	1 3/4	NCI	29	1	40	Rush	90	Female	5	AD	14	5
16	U KY	81	Male	2 3/4	NCI	29	2	41	Rush	89	Female	5 2/4	AD	10	3
17	U KY	92	Female	8	NCI	29	2	42	Rush	89	Female	5 2/4	NCI	28	4
18	U KY	68	Female	7 2/4	AD	17.5	6	43	Rush	87	Female	3 2/4	NCI	30	3
19	U KY	87	Male	7 3/4	AD	18	6	44	Rush	95	Female	5 1/4	NCI	30	3
20	U KY	91	Female	7 2/4	AD	23.5	5	45	Rush	91	Female	5	NCI	29	2
21	U KY	82	Female	6 1/4	AD	19	6	46	Rush	91	Female	8 1/4	AD	23	4
22	U KY	84	Male	1 2/4	NCI	28	2	47	Rush	81	Female	5 3/4	NCI	28	2
23	U KY	65	Female	4 1/4	AD	3	6	48	Rush	77	Female	5 3/4	NCI	30	1
24	U KY	68	Male	8	AD	13	5	49	Rush	85	Female	3 2/4	NCI	29	4
25	U KY	84	Male	5 1/4	AD	13	6	50	Rush	92	Female	5	AD	22	5
								51	Rush	85	Female	4 3/4	NCI	28	4
								52	Rush	92	Female	3	NCI	29	4
								53	Rush	76	Female	3	NCI	29	1
								54	Rush	96	Female	4 1/4	NCI	26	3

^a^Sources: U KY: University of Kentucky Alzheimer’s Disease Center, Lexington, KY; Rush: Rush Alzheimer’s Disease Center, Rush University, Chicago, IL

^b^PMI; Post-mortem interval, rounded to nearest quarter hour

^c^NCI: non-cognitively impaired; AD: Alzheimer’s disease

^d^MMSE: Mini-mental state exam score

### RNA extraction

RNA was extracted from frozen tissue using a modified Ambion PureLink mini kit protocol (#12183018A). Briefly, between 10 and 25 mg of tissue was placed in a 2 ml round bottom tube. One milliliter of Trizol (ThermoFisher #15596026) was added. Tissue was sonicated on ice until homogenous, and was allowed to incubate for 5 min at room temperature. Then 200 μl of chloroform was added, and the sample was vortexed for 15 s. Following a 3 min incubation at room temperature, the samples were centrifuged at 12,000 × *g* for 15 min at 4 °C. The upper aqueous layer was transferred to a clean 1.5 ml tube, and an equal volume of 70% ethanol was added. The sample was vortexed and then processed following the manufacturer’s instructions. RNA was eluted in a final volume of 50 μl of nuclease free water, and was then quantified to be used as a template for cDNA synthesis.

### MicroRNA quantification by qRT-PCR

Quantitation of miR-298 levels was determined using two methods as described below. miR-298 levels in human tissue were analyzed by qPCR using both relative and absolute quantitative techniques. For relative quantitation, a probe-based assay for miR-298 (TaqMan 002190) was measured and compared with the control small RNA RNU48 (TaqMan 001006 labeled with VIC) [56]. Briefly, template for qPCR was generated using the TaqMan microRNA reverse transcription kit (Applied Biosystems 4366596) following the manufacturer’s recommended protocol with an input of 10 ng of RNA. qPCR was performed on an ABI 7500 instrument in 20 μl reactions, which were incubated for 40 amplification cycles. Each reaction contained 1.3 μl of reverse transcription product as template, 2× master mix minus uracil-N-glycosylase (UNG) Applied Biosystems 444040), and each of the TaqMan assays listed above. Ct values were determined using a constant threshold, and fold change was calculated by the delta-delta Ct method.

Human hsa-miRNA-related reagents were procured commercially. The lyophilized and frozen powder was dissolved in RNase-free water, and the concentration and purity was checked by NanoDrop (ThermoFisher). Dharmacon (Lafayette, CO) supplied miRIDIAN microRNA mimics are double-stranded RNA oligonucleotides designed to supplement endogenous microRNA activity and the mature miRNA sequence is shown below: hsa-miR-298—AGCAGAAGCAGGGAGGUUCUCCCA. (Dharmacon, Cat# C-301212–01–0005); hsa-miR-298 inhibitor—the sequence is proprietary of Dharmacon (Cat# IH-301212–02). Negative control mimic 2 (NCM2)—UUGUACUACACAAAAGUACUG. (*Caenorhabditis elegans* miR-239b) (Dharmacon, Cat# CN002000–01–05).

For absolute quantification, the TaqMan Advanced cDNA synthesis kit (Applied Biosystems A28007) was used to produce template for qPCR. Ten ng of RNA was polyadenylated, ligated to an adapter, reverse transcribed, and amplified, resulting in cDNA capable of being interrogated with any TaqMan Advanced miR assay. Then qPCR amplification reactions were assembled including 2 μl of miR-AMP product as template, 2× PrimeTime master mix (Integrated DNA Technologies 1055772), and TaqMan Advanced assay miR-298 (Applied Biosystems 478430) in a total of 10 μl. The reactions were subjected to 40 rounds of amplification in an ABI 7500 thermocycler. A standard curve of not less than five data points was created using known concentrations of an miR-298 synthetic oligonucleotide (IDT, Coralville, Iowa). Ct values were determined using a constant threshold. Construction of the standard curve was performed by creating a scatter plot in Excel based on the Ct values of the samples of synthetic miR-298. The *x*-axis of the plot was converted to log scale, and a logarithmic trend line was fitted to the standards. The equation of the slope and the R squared value was displayed on the graph. Concentrations of unknown samples were determined by extrapolation using the slope equation generated by the standard curve.

### Generation of mice overexpressing miR-298

AAV9 vector plasmids containing an expression cassette consisting of a human elongation factor-1α promoter followed by miR-298 or mock sequence and human cytomegalovirus promoter followed by cDNA encoding GFP, were provided by SignaGen Laboratories (Rockville, MD). A viral load of 10^11^ vector genomes of each of these constructs was injected into the tail veins of 5 weeks old C57Bl6 mice, as previously published [57]. The study was carried out in accordance with the National Institute of Health Guide for the Care and Use of Laboratory Animals and was approved by the National Institute of Neurological Disorders and Stroke Animal Care Committee. All experiments were conducted blindly by third party concealment of treatments with individually uniquely coded vials. Animals were assigned to treatment groups via Research Randomizer [58].

### Preparation of mouse spinal cord lysates

Lysates from spinal cords were denatured at 95 °C in 1 × LSB and processed for SDS–PAGE. For measuring tau protein, samples were loaded as pseudoreplicates and the average value of pseudoreplicates from each sample was averaged and provided a data point shown. For APP and BACE1, samples were loaded as single replicates and are from the same western blot. Proteins were normalized to Ponceau stain values obtained from a region at a similar molecular weight as each protein of interest.

### Alzheimer’s Disease Neuroimaging Initiative (ADNI) phenotype and biomarker determination

Inclusion and exclusion criteria, clinical and neuroimaging protocols, and other information about ADNI have been published previously [59] and can be found at www.adni-info.org. Demographic information, whole-genome sequencing data, neuropsychological test scores, and diagnostic information are publicly available from the ADNI data repository (http://www.loni.usc.edu/ADNI/). Baseline CSF samples were obtained using previously [60] reported methods for three CSF measurements (Aβ42, total tau (t-tau), and tau phosphorylated at the threonine 181 (p-tau_181p_)).

### ADNI whole-genome sequencing (WGS) analysis

WGS was performed [61] on blood-derived genomic DNA samples obtained from 817 ADNI participants. Samples were sequenced on the Illumina HiSeq2000 using paired-end read chemistry and read lengths of 100 bp (www.illumina.com). The resulting qseq files were converted into fastq files, (flat text for Phred). An established next-generation sequencing analysis pipeline based on Genome Analysis Toolkit (GATK) was used [61]. Quality checks and read statistics were performed on raw sequence data using FastQC. Short-read sequences were mapped to the NCBI reference human genome (build 37.72) using the Burrows–Wheeler Aligner, allowing for up to two mismatches in each read. During the alignment, we used only bases with Phred Quality >15 in each read to soft clip low-quality bases, retain only uniquely mapped pair-end reads, and remove potential PCR duplicates. Local realignment of any suspicious reads after initial alignment further refined results. Reported base calling quality scores obtained from the sequencer were recalibrated to account for covariates of base errors, such as sequencing technology and machine cycle.

Realigned reads were written to a BAM file for further analysis to identify all variants with statistical evidence for an alternate allele present among samples using GATK HaplotypeCaller for multi-sample variant callings [62]. For variants that passed recommended variation quality criteria, ANNOVAR was used to annotate all variants’ single nucleotide polymorphisms (SNPs) and short insertion/deletions (indels). We performed standard quality control procedures in WGS to assess quality and remove individuals and genetic variants with poor quality. We excluded variants that did not pass variant quality score recalibration step using GATK in the WGS analysis pipeline, and we removed variants with genotype quality scores < 20. Quality of variant calls was assessed by comparing sequencing-derived SNPs with those obtained from the Illumina Omni 2.5 M genotyping array in order to estimate the concordance rate for each individual.

Furthermore, in order to prevent spurious association due to population stratification, we selected only non-Hispanic Caucasian participants (*N* = 757; 259 cognitively normal controls, 219 early MCI, 232 late MCI, 47 AD cases), which clustered with HapMap CEU (Utah residents with Northern and Western European ancestry from the CEPH collection) or TSI (Toscani in Italia) populations using multidimensional scaling (MDS) analysis (www.hapmap.org) [63].

### Statistical analysis

Generalized linear models (glm) and analysis of deviance (ANOVA) were used where applicable, followed by pairwise comparisons of estimated marginal means via false discovery rate-adjusted *p* values [64]. In the case of pseudoreplicates, generalized linear mixed-level models were substituted for glm. Using all WGS-identified SNPs in the *MIR298* gene region, we performed an SNP-based association analysis using a linear regression. Potential confounding factors (age, sex, and batch) for CSF measurements were tested as covariates. The glm relaxes assumptions conventional linear models, such as gaussian distribution and homoscedasticity. Variances are not required to be similar between groups.

## Results

### Overview of study design

We employ a comprehensive and integrated strategy of in silico identification, Bioinformatics prediction and followed by the Validation, Functional, Correlation, Association and Interspecies studies (Fig. 1). In short, we present convergent evidence in support for the proposed miR-298 effect, from human cell culture transfection and protein studies, human genetics, human brains, and animal model brains, as shown in subsequent figures.

### Identification of miR-298 as a putative regulator of APP, BACE1, and MAPT

**Fig. 2:**
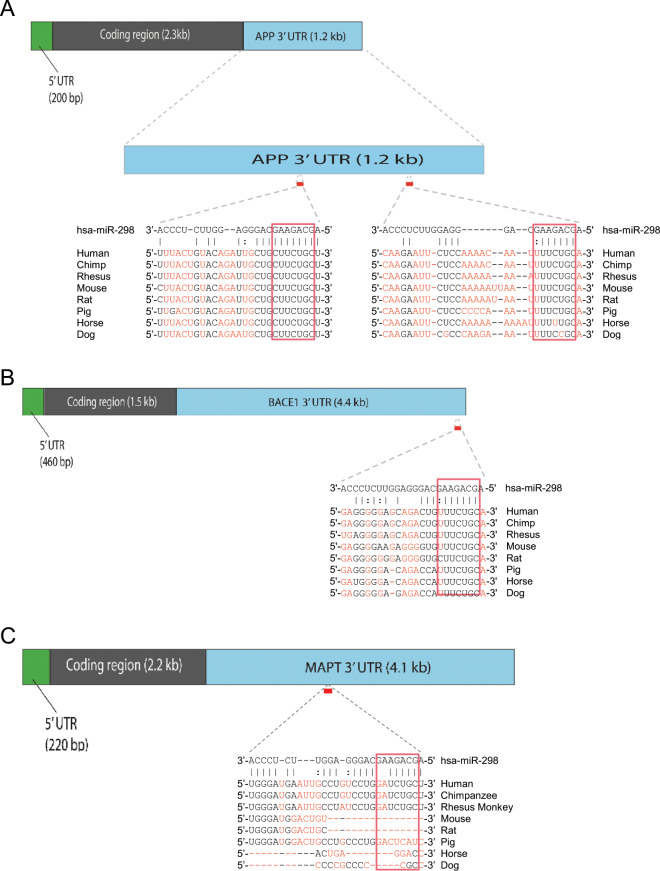
Predicted binding sites of miR-298 on 3′-UTR sequences. **a** APP. **b** BACE1. **c** MAPT. Multiz alignments [52] from the UCSC genome database [51] of binding sites on human 3′UTR vs. other mammalian sequences are also shown. The predicted miR-298 affinity with MAPT would most likely operate via noncanonical binding.

miR-298 is predicted to target the 3′ UTR of both APP and BACE1, according to multiple bioinformatic predictive algorithms (Table 2). The highly negative ΔΔG [47] values of −14.24 for the APP 3′-UTR and −13.22 for the BACE1 3′ UTR predict strong binding efficiency between miR-298 and the APP and BACE1 UTRs. PhastCons [65, 66] scores suggest that target sites for miR-298 on the UTRs are well-conserved. Whereas, the context ++score percentile [67] suggests that the predicted miRNA sites on both UTRs are highly reliable. For the APP 3′-UTR, miR-298 has two possible target sites: A 7-mer-m8 target sequence and a 7-mer-A1 sequence (Fig. 2a). Both these target sites are highly conserved across the seed sequence, with weaker homology for the rest of miR-298. miR-298 has one 7-mer-A1 predicted site on the BACE1 3′-UTR (Fig. 2b) which is also well-conserved across species.

**Table 2:** miR-298 predictions of interaction with APP and BACE1 3′-UTRs.

Algorithm	Score^a^	
	APP	BACE1
TargetScanHuman 7.1 ^43b^	83	79
PicTar ^44c^	NA	NA
DIANA-microT ^45d^	0.871	0.644
microRNA.org ^46e^	0.7359	0.6359
PITA ^47f^	−14.24	−13.22
rna22 ^48g^	−20.9	−12.1

^a^None of these utilities indicated interaction between the canonical miR-298 seed sequence and the tau 3′-UTR

^b^Context ++score percentile is a rank of affinity between a given miRNA and target sequence vs. other miRNAs in the database

^c^PicTar could not scan miR-298. It is not conserved across mammalian species

^d^miTG score is weighted sum of the scores of all identified MREs (hidden Markov model) on the 3′-UTR

^e^PhastCon score is based on conservation of target site (hidden Markov model)

^f^ddg score is based on hybridization energy and site accessibility

^g^Folding energy (kcal/mol), allowing for a single G:U bulge

After performing an analysis of association between known miR-298 associated SNPs and AD endophenotypes, (See “Single nucleotide polymorphisms” section), we discovered that SNP rs6070629 is significantly associated with elevated phospho-tau in CSF. Bioinformatics tools such as TargetScan, microRNA.org, and PITA did not predict miR-298 as targeting MAPT. Therefore, we searched for noncanonical binding sites using RNAhybrid 2.2 [50, 68] and found a possible miR-298 binding site on the MAPT 3′-UTR (Fig. 2c). This site is not conserved in rat or mouse MAPT 3′-UTRs, but it is conserved in rhesus monkey sequence.

### miR-298 targets both the APP 3′-UTR and BACE1 3′-UTR in human glial cell cultures

**Fig. 3:**
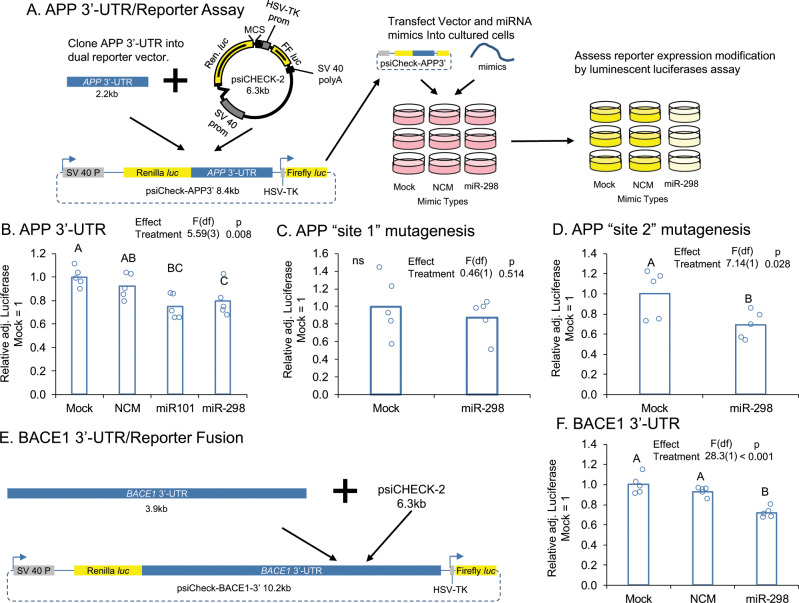
miR-298 targets the APP and BACE1 3′-UTR. **a** The full-length APP 3′-UTR was cloned into psiCheck 2 (Promega), which contains both *a Renilla* luciferase gene, fused to the APP 3′-UTR in our experiment, and an internal control firefly luciferase gene. The fusion vector was transfected along with miRNA mimics and controls into cultured cells. When miRNA was active with the APP 3′-UTR, luminescence was reduced vs mock-transfected cultures. **b** Transfection of miR-298 in HeLa cells reduces APP 3′-UTR-mediated luciferase expression. **c** Mutation of target site-1 does not reverse this effect. **d** Mutation of site 2 reverses the effect. **e** The full-length BACE1 3′-UTR was cloned into psiCheck 2 and transfected into cell cultures along with miR-298 and control oligomers. **f** miR-298 also reduces BACE1 3′-UTR-mediated luciferase expression.

Co-transfection in U373 cells of miR-298 with a reporter vector containing the full-length APP 3′-UTR (Fig. 3a) reduced luciferase expression by 25% compared with the mock-treated cultures (Fig. 3b). miR-101, a previously validated [28] microRNA capable of binding to the 3′-UTR was used as a positive control and reduced luciferase expression by 30%. NCM did not significantly change luciferase expression. To establish target specificity of miR-298, the two target sites on the APP 3′-UTR were mutagenized separately. Mutagenesis of target site 1 did not reverse miR-298-mediated targeting of the APP 3′-UTR (Fig. 3c), whereas mutagenesis of target site 2 reversed miR-298’s effects (Fig. 3d) on luciferase expression.

In HeLa cells, we also showed that co-transfection of miR-298 with a luciferase reporter containing the full-length BACE1 3′-UTR (Fig. 3e) reduced reporter expression by 25% compared with the mock treatment group (Fig. 3f). An NCM not predicted to target the human BACE1 3′-UTR did not reduce luciferase activity compared with mock alone (Fig. 3f).

### miR-298 inhibitor reversed miR-298-mediated APP and BACE1 reduction in a mixed primary human culture

**Fig. 4:**
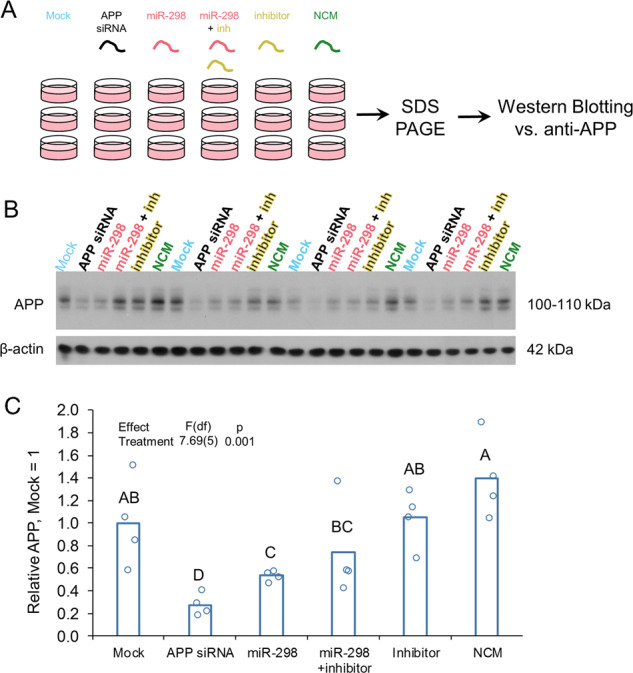
miR-298 inhibitor partially reverses miR-298-mediated reduction of APP protein in human primary brain culture. **a** Primary human mixed-brain cell cultures were mock-transfected or transfected with APP siRNA, miR-298, miR-298 inhibitor (antisense to miR-298), miR-298 plus inhibitor, or NCM. Cells were cultured 72h  after transfection, lysed, and cell lysate proteins were analyzed on SDS–PAGE followed by western blotting for APP. **b** Reduction by miR-298 is less severe than that imposed by APP siRNA. *n* = 4. Pairwise differences (*p* ≤ 0.05) are indicated by letters. Treatments sharing a letter did not significantly differ. **c** The addition of miR-298 inhibitor reverses miR-298-mediated reduction in APP levels.

**Fig. 5:**
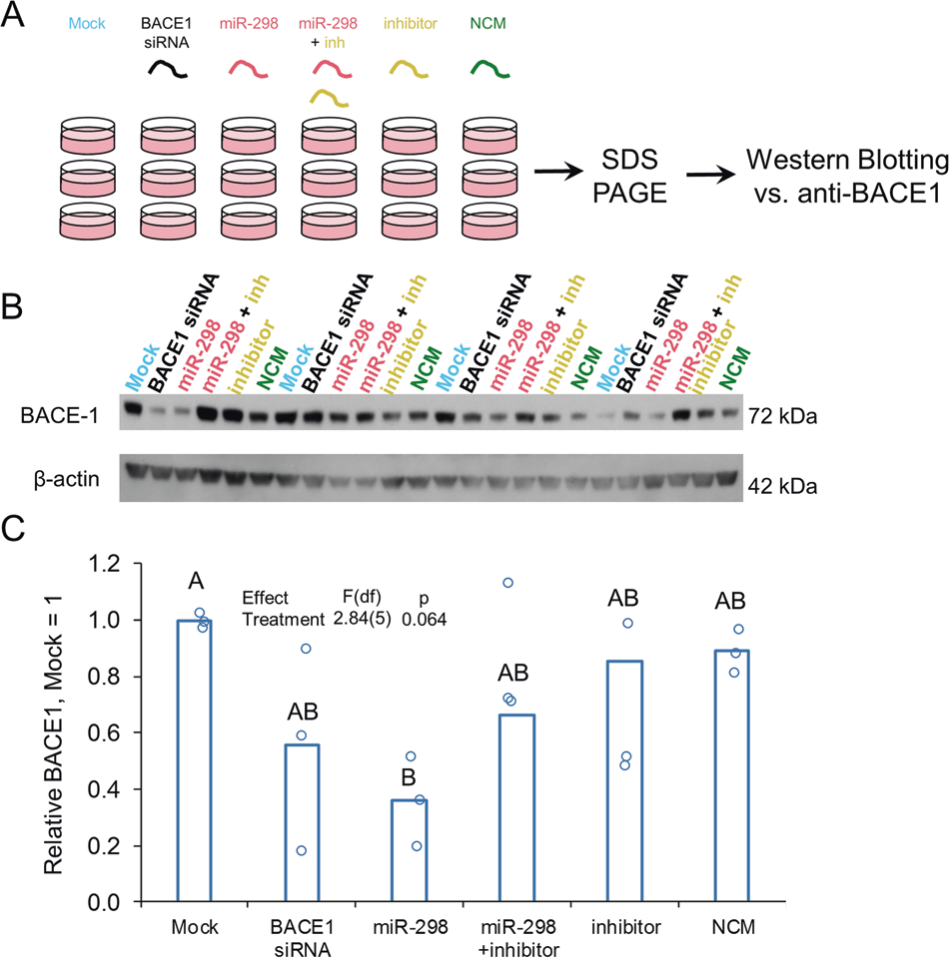
miR-298 inhibitor reverses miR-298-mediated reduction of BACE1 protein in human primary brain culture. **a** Primary human mixed-brain cell cultures were mock-transfected or transfected with APP siRNA, miR-298, miR-298 inhibitor, miR-298 plus inhibitor, or NCM. Cells were cultured further after transfection, lysed, and lysate run on SDS–PAGE followed by western blotting for BACE1. **b** Western blot of BACE1 levels. **c** The Addition of miR-298 inhibitor reverses effect of miR-298 on BACE1 levels. *n* = 4, pairwise differences (*p* ≤ 0.05) are indicated by letters. Treatments sharing a letter did not significantly differ.

By transfecting a mixed, primary human brain culture developed in our lab [55] with miR-298 plus inhibitor oligomers (Fig. 4a), we showed that miR-298 overexpression reduced APP expression by 40%, whereas co-transfection of an miR-298 antagomir partially reversed this effect (Fig. 4b, c). The inhibitor alone had no effect on APP expression, nor did a negative control miRNA mimic (Fig. 4b, c). APP siRNA was used as a positive control and knocked down APP expression by 75%. Likewise, BACE1 expression in the same mixed, primary human brain culture (Fig. 5a) was reduced by miR-298 (Fig. 5b, c) compared with mock-transfected cells. Co-transfection of the antagomir reversed miR-298-mediated reduction of BACE1 (Fig. 5b, c).

### miR-298 alters APP levels in a dose-responsive and time-sensitive fashion

**Fig. 6:**
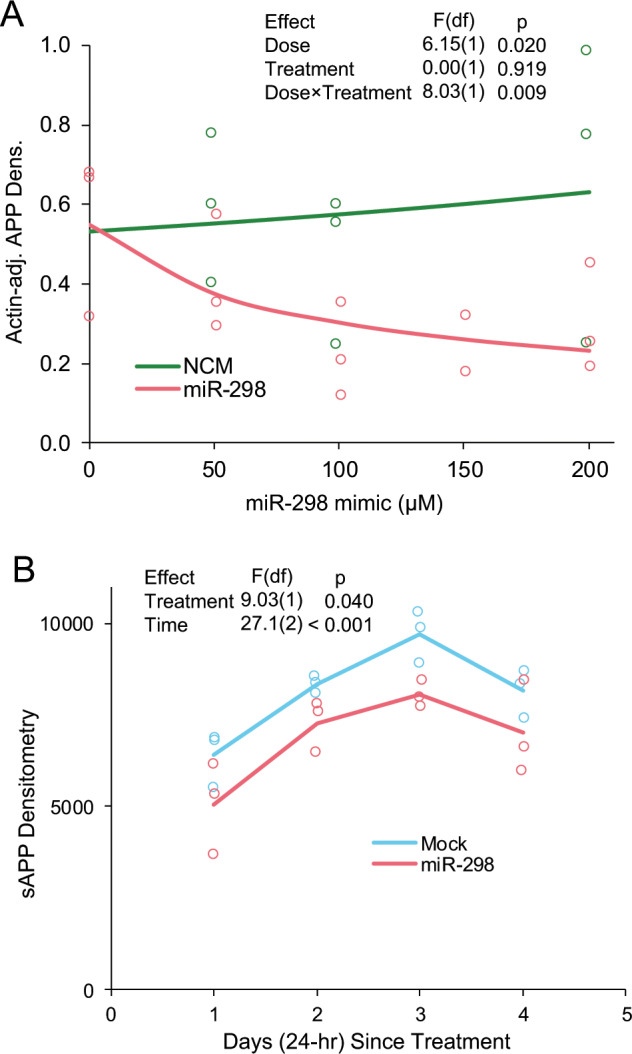
miR-298 alters APP levels in human mixed-brain culture in dose- and time-dependent fashions. **a** Human mixed-brain culture was treated by doses of miR-298 and NCM at doses of 0, 50, 100, 150, and 200 µM for miR-298 and at 0, 50, 100, and 200 µM for NCM. Cells were lysed and subject to western blot as described herein and visualized blots scanned for densitometry. For all doses, *n* = 3. **b** Human mixed-brain cultures were treated with a single dose (150 µM) of miR-298 or with transfection reagent containing no miRNA mimic (Mock). Culture conditioned media was harvested at 1, 2, 3, and 4 days. sAPP was measured by western blot followed by densitometry. For all time points, *n* = 4.

We used cell lysates to study the effect of miR-298 on APP at various doses and conditioned media to examine the time-course kinetics of miR-298 activity, by western immunoblotting. Levels of total APP densitometry, adjusted by β-actin were significantly reduced by increasing dose vs no significant reduction for NCM-treated cells (Fig. 6a). In addition, the effect of miR-298 dose leveled off in a nonlinear fashion.

Levels of sAPP in miR-298-treated cell conditioned media were significantly reduced compared with mock-transfected cells across the time series of 1–4 days (Fig. 6b). A further confounding element was that both sets of cultures (treated and untreated) produced sAPP at an ascending then decreasing rate, which we “modeled out” by treating time as a 2nd-order polynomial. Notably, both time and miRNA treatment were significant effects, but miRNA treatment did not alter the trajectory of sAPP production over time. These results were consistent with results obtained from a western blot using cell lysates measuring intracellular (and membrane-bound) APP (data not shown).

### miR-298 treatment reduced levels of soluble Aβ species as well as soluble APP (sAPP)

**Fig. 7:**
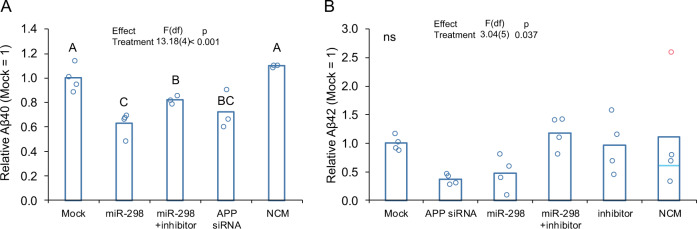
miR-298 results in reduction of Aβ40, Aβ42 in human mixed-brain culture. Cultures were treated with miRNA mimics or inhibitors and levels of (**a**) Aβ40 peptide (pairwise differences, p ≤ 0.05, are indicated by letters. Treatments sharing a letter did not significantly differ.) and of (**b**) Aβ42 peptide in conditioned media measured. No pairwise differences were significant for Aβ42, indicated by "ns". Red point indicates a statistical outlier, dashed line for "NCM" indicates mean excluding outlier. Significance for model and pairwise significances were not altered by excluding outlier from analysis. A-B: *n* = 4, *p* < 0.05.

In conditioned media from transfected wells, miR-298 reduced significantly levels of both Aβ40 and Aβ42 peptides (Fig. 7). Consistent with western blot results with APP and BACE1, coadministration of the antagomir to miR-298 reversed miR-298-mediated reduction of both Aβ40 and Aβ42 peptides (Fig. 7).

### miR-298 altered levels of two specific isoforms of tau

**Fig. 8:**
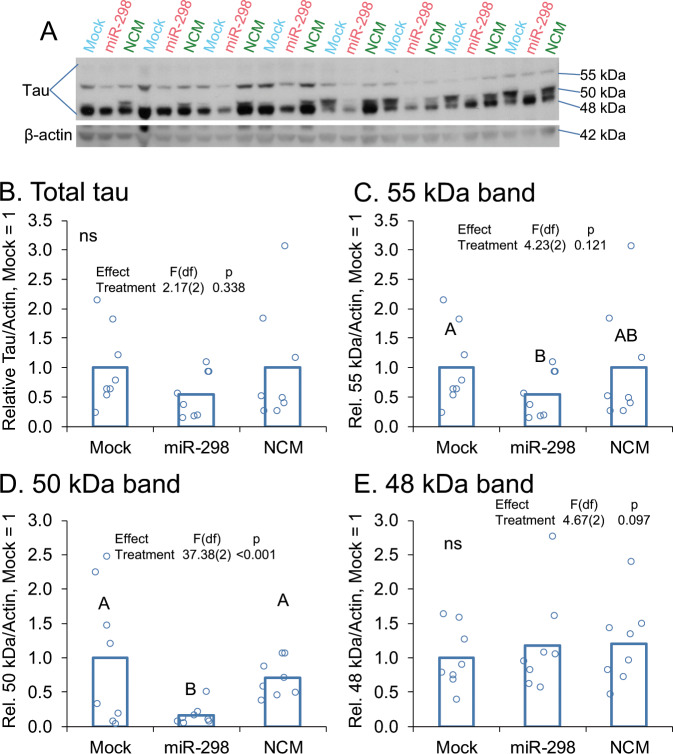
miR-298 reduces two specific isoforms of total tau protein. **a** Western blotting from a mixed-brain cell type human primary culture is shown. (*n* = 4). **b** Total tau showed no significant differences by treatment. **c** Treatment with miR-298 associated with a significant reduction of a ~55 kDa band. **d** Treatment with miR-298 also produced significant differences for a 50 kDa band. **e** Treatment with miR-298 produced no significant differences for a 48 kDa band. Pairwise differences (*p* ≤ 0.05) are indicated by letters. Treatments sharing a letter did not significantly differ.

Using lysates obtained from mixed, primary human brain cultures [57], we tested possible modulation of MAPT levels by miR-298, and we observed that miR-298 did not significantly alter overall tau levels (Fig. 8b). However, three potential tau isoforms appeared by western at 55 (Fig. 8a, c), 50 (Fig. 8a, d), and 48 kDa (Fig 8a, e) approximate MW. When each band was quantified separately, we noticed that the 55 kDa and 50 kDa bands were significantly reduced following miR-298 treatment vs. mock treatments (Fig. 8c). β-actin was used as a loading control and was not changed significantly across any groups. We should mention that several other smaller bands (<48 kDa) also appeared in our blot, but these were excluded from analysis as we wished to constrain ourselves to sizes known to associate with tau protein.

### In vivo overexpression of human miR-298 resulted in nonsignificant reduction of APP, BACE1, and tau proteins but elevations of corresponding mRNA levels in mice

Using spinal cord lysates obtained from experiments described previously [57], we measured the expression of APP, BACE1, and the 55 and 50 kDa isoforms of tau from the brains of control and miR-298 overexpressed mice. We found that miR-298 overexpression did not correspond to significant differences in levels of any of the proteins measured, although a trend for reduction was present in all three proteins (Supplementary Fig. 1).

### Human miR-298, but not mouse miR-298, negatively regulates endogenous APP and BACE1 in human cells

**Fig. 9:**
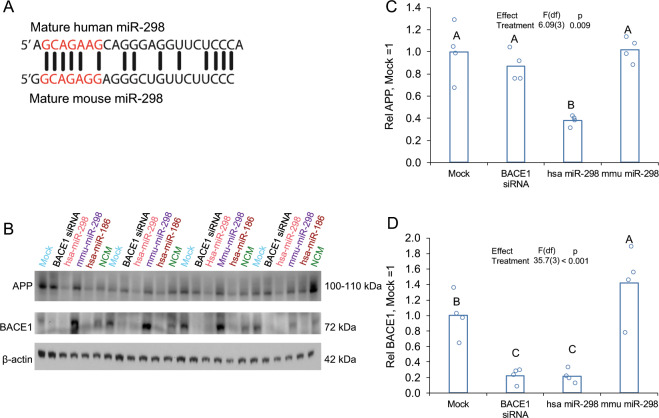
Human miR-298, but not mouse miR-298, reduces APP and BACE1 expression in human glioblastoma cells. **a** Homology between mature hsa-miR-298 and mmu-miR-298. **b** Western blot of miR-298 reduction of APP and BACE1. β-actin served as a loading control. Densitometry of **c** APP and **d** BACE1 normalized to β-actin. *n* = 4, Pairwise differences (*p* ≤ 0.05) are indicated by letters. Treatments sharing a letter did not significantly differ. Human miR-186 was included as a human miRNA not expected to alter APP levels (β-actin-adjusted) in U373 cells.

Although there is limited homology between the sequences of mature human miR-298 (hsa-miR-298) and mouse miR-298 (mmu-miR-298), seed sequences of miR-298 in the two species have a perfect complementarity except for one nucleotide at the 6th position from the 5′ end of the start of the seed sequence (Fig. 9a).

Transfection of hsa-miR-298 into human U373 cells reduced APP expression by 40% (Fig. 9b, c), compared with mock-transfected cells, whereas neither BACE1 siRNA nor mmu-miR-298 changed APP expression significantly (Fig. 9b, d). Hsa-miR-298 overexpression and administration of BACE1 siRNA reduced BACE1 expression by 60% and 80% respectively. Mouse miR-298 did not have a significant effect on APP expression (Fig. 9c, *p* > 0.05); however, an increase in BACE1 expression was observed (Fig. 9d, *p* < 0.05).

### miR-298 levels vary in AD only in the temporal lobe

**Fig. 10:**
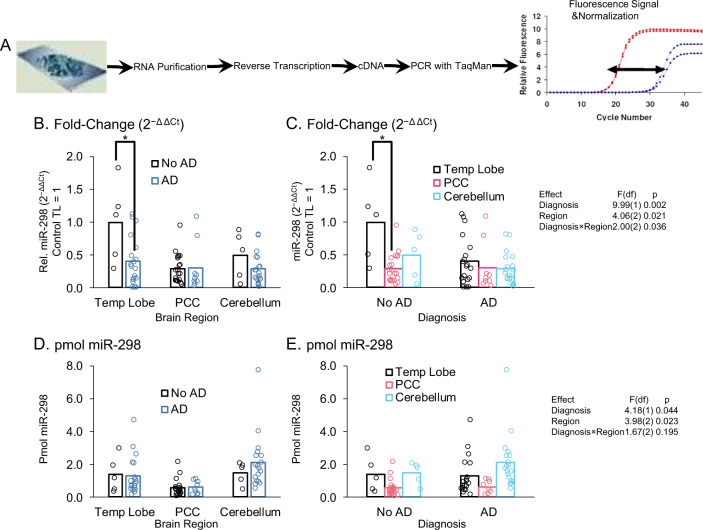
Levels of miR-298 in control vs. AD brain regions. **a** RNA was purified from frozen human brain tissues as described herein. RNA was then subject to reverse transcription and PCR with TaqMan kits. Signal was normalized to RNU48 signal. **b** Comparing non-AD vs. AD levels by brain region. Only TL has an AD-related difference (*p* < 0.05). **c** Comparing brain regions in non-AD and in AD subjects. PCC has a significantly (*p* < 0.05) lower level of miR-298 only in non-AD subjects. **d**, **e** miRNA measured by absolute quantification. No significant differences.

We measured levels of miR-298 in TL, PCC, and cerebellum from brains of both AD patients and age-matched non-AD controls (Fig. 10a). We used the 2^−Δ ΔCt^ method, adjusted to non-AD TL = 1 (Fig. 10b, c) and absolute quantification (Fig. 9d, e). When examining each region separately (Fig. 10c), miR-298 was significantly (*p* < 0.05) reduced in TL only. Notably, recently acquired miRNA sequencing data demonstrated a significant ~3-fold upregulation of miR-298 in the PCC of AD compared with non-AD subjects (non-AD [*n* = 10]: 0.65 ± 0.26 [mean ± S.D.]; AD [*n* = 10]: 1.66 ± 0.5; *p* < 0.0001). However, qPCR-based query of PCC samples did not return significant differences (Fig. 10c). When we looked at the three brain regions, separated into non-AD and AD, the only significant difference was that TL in non-AD subjects was significantly (*p* < 0.05) higher than PCC. Although non-AD TL levels were higher than non-AD cerebellum levels, this difference was not significant. In short, the only apparent effect of AD we observed was reduction of miR-298, measured by 2^−ΔΔCt^ method, in the TL. However, differences were not significant if looking at miR-298 levels by absolute quantification.

### Single nucleotide polymorphisms (SNPs) in the miR-298 gene associated with AD-related CSF biomarkers

**Fig. 11:**
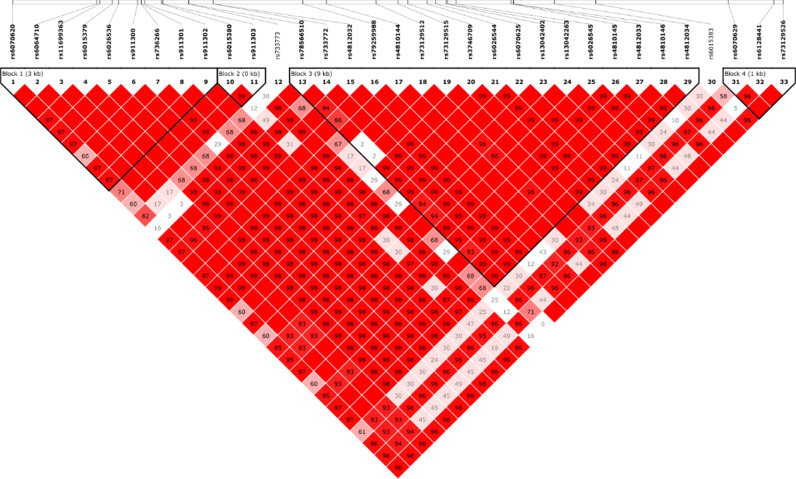
Identification of relevant SNPs within the *MIR298* gene boundary. LD blocks for region proximal to *MIR298* gene reveals four blocks of SNPs. The top thick line represents a strand of a chromosome. The white bars on the blue line of the chromosome are SNPs (single nucleotide polymorphisms) that have been identified and sequenced. These SNP locations or loci are labeled in this picture standard nomenclature. Each of these SNPs has a name that has the format rs#…# where “#…#” is a numeric code of varying length. Each SNP is represented by a labeled gray triangle below the thick blue line (the chromosome). We identified 33 common SNPs (minor allele frequency (MAF) ≥ 0.05) within ±10 kb from the *MIR298* gene boundary. Of the 33 SNPs, 32 exist within four LD (linkage disequilibrium) blocks of 2, <1, 9, and 1 kb in length.

**Fig. 12:**
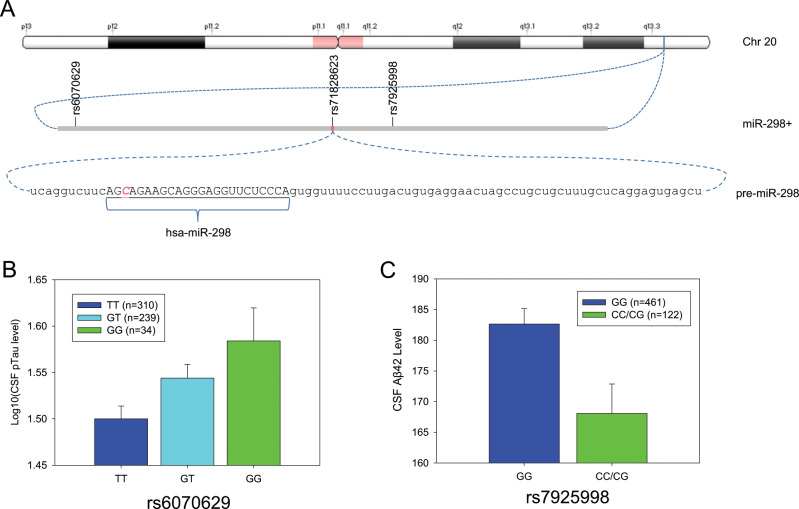
SNPs proximal to *MIR298* gene with AD-associated phenotypes. **a** *MIR298* resides on chromosome 20, in band q13.3. Two potentially functional SNPs, rs6070629, and rs7925998, are at ~8.4 kb upstream and 1.9 kb downstream of the *MIR298* gene. An apparently nonfunctional SNP (rs71828623) lies within the miR-298 seed sequence. The 88-base pre-miR-298 contains the mature miR-298 sequence. Position of the nonfunctional rs71828623 is indicated. **b** SNP rs6070629 associated with elevation of CSF levels of a specific phosphorylated τ in a dose-dependent manner. The minor allele (“G”) associates with elevated p-τ levels. **c** SNP rs7925998 associated with reduction of CSF Aβ42. The presence of either one or two minor “C” alleles of rs7925998 associated with a reduction of CSF Aβ42.

We identified 33 common SNPs (minor allele frequency (MAF) ≥ 0.05) within ±10 kb from the *miR-298* gene boundary (Fig. 11) from whole-genome sequencing data. We performed linkage disequilibrium (LD) analysis. LD is a nonrandom association of two sequence features, such as SNPs. Mapping multiple LD events can also reveals haplotype blocks. Of the 33 SNPs, 32 exist within four LD blocks (Fig. 11) of 2, <1, 9, and 1 kb in length. Two of these SNPs (Fig. 12a, rs6070629, rs79259988) at 8.4 kb upstream and 1.9 kb downstream of the pre-miR-298 coding sequence, respectively, associated with AD biomarkers. The minor allele of SNP rs6070629 is relatively common (25.8 ± 2.6% in reported frequency). Subjects carrying the minor “G” allele of the SNP have higher CSF τ181p (τ phosphorylated at threonine 181), in a dose-dependent fashion (*β* = 0.045, *p* = 0.0048) GG>GT>TT (Fig. 12b). SNP rs79259988 (8.9 ± 0.8% frequency) in miR-298 is nonsignificantly associated with CSF Aβ42, specifically the minor allele associated (*β* = −12.41, *p* = 0.160) with reduced CSF Aβ42 level (Fig. 12c). We found one SNP (rs751823623) specifically within the miR-298 seed sequence, but it had no association with AD, and the minor allele was extremely rare (<0.0001%).

## Discussion

Although it remains controversial, the amyloid hypothesis [6] suggests that Aβ generation is a causative event in the onset of AD. Therefore, the reduction of the potentially toxic Aβ peptide remains a viable therapeutic goal. While the peptide could be reduced by other means, such as targeting various Aβ-degrading enzymes [69], we focused on reducing generation of Aβ. Drug therapies targeting APP and BACE1 have had limited success due to factors including effects of drugs on organs other than the brain [11] as well as the limitation of specificity. It is difficult to produce drugs that would target both crucial proteins involved in the generation of Aβ; namely APP and BACE1. The other dominant hypothesis of AD etiology is that it is primarily due to intracellular accumulation of p-tau [70]. A drug target that modulates all three of these potential participants in AD could be highly impactful. MicroRNAs are short, endogenous, single-stranded RNA that serve as a “socket” for RISC regulation of mRNA translation. Therefore, the identification of an miRNA that targets both APP and BACE1 would be advantageous in the reduction of Aβ. In addition, accumulation of hyperphosphorylated tau is a hallmark of AD and multiple other tauopathies. An miRNA that might happen to regulate, even in part, both Aβ and tau could potentially be quite useful for therapeutic development.

Previous work has shown miR-298’s role in targeting proteins implicated in cancer, such as catenin delta 1 [71], apoptotic protein Bax [72], and polycomb protein enhancer of zeste [73]. MiR-298 has been shown to improve outcomes in a spinal muscle atrophy model [57] and to paradoxically show worsening of spinal cord injury outcomes in murine models [74]. Mouse miR-298 targets mouse BACE1 [42], but effects of human miR-298 on human BACE1 are unknown.

Using a multitude of approaches (Fig. 1), we found that miR-298 regulates protein expression of APP, BACE1, and at least two isoforms of tau. Initially, we found that miR-298 was a putative modulator of both BACE1 and APP using bioinformatics algorithms. We confirmed that miR-298 targets the APP 3′-UTR via one of the two predicted target sites. The putative seed site of miR-298 on BACE1 3′-UTR was not mutagenized to test its validity. Although the presence of a predicted miRNA binding site strongly favors that the mechanism is direct regulation through conventional RISC mechanisms, it always remains a (however distant) possibility that miR-298-mediated reduction of BACE1 would be through an unidentified and entirely speculative indirect mechanism. We also confirmed that miR-298 reduces APP and BACE1 protein expression in two different cell cultures.

Murine overexpression of miR-298 in vivo did not produce a statistically significant alteration in APP, BACE1, or t-tau protein or mRNA levels. While this result does *prima facie* disagree with our results obtained in tissue cultures, it is important to note that all three proteins showed nonsignificant reductions and therefore we believe that a larger sample size may result in more statistically robust reductions. Thus, we infer that cross-species interaction may be weaker, not absent. We reiterate that human miR-298 and the host mouse miR-298 sequences differ. We used spinal cord because the concurrently discovered SNP variations used CSF markers, and it was possible they reflected spinal cord differences. We included the “negative” mouse data to illustrate limitations of using animal models in AD research. Human systems (both the sequence and models) must be used to verify animal results, and a negative animal result does not necessarily entail a negative human result, particularly given the differences between mature hsa-miR-298 and mmu-miR-298 sequences. Cross-species difference that may not be differences in the specific “target site” can produce different results. We had chosen the specific hsa-miR-298 viral delivery due to its efficacy in reducing levels of androgen receptor in mouse models [57], which we presumed would apply to other mouse mRNAs’ translation vs miR-298. Our data suggest that such presumptions of success (or failure) should be treated with caution. Nevertheless, we found convergent supporting evidence for the proposed miR-298 effect, from human cell culture, human genetics, human brains, and animal model nervous system tissue. A case can be made that this molecule deserves more attention and study [75].

In any case, to find an explanation for the negative result in mice, we tested mouse miR-298 vs. human miR-298 in human-derived cell cultures. The mouse miR-298 did not reduce APP and BACE1 protein levels, while human miR-298 was active. We noted poor conservation between miR-298 sequences of both species. It is also worthwhile to note that *secondary structure* of a given mRNA exerts a significant effect upon miRNA activity [76]. Comparison of the mouse and human APP 3′-UTR sequences, for example, revealed that the mouse sequence was over 100 bases shorter (data not shown), which would reasonably produce important secondary structure differences.

When we examined known SNPs of hsa-miR-298, we found an AD endophenotype associated with specific tau phosphorylation. To capitalize on this serendipitous discovery from clinically derived data, we tested miR-298’s role on tau levels. We used lysates from our primary human mixed culture and found that miR-298 modulated a specific (55 kDa) form of tau, but not t-tau or the major isoform (48 kDa). The identification of the tau-immunoreactive 55 and 50 kDa bands are unclear, but may represent specific tau isoforms or phosphorylated tau moieties. The tau protein can be alternatively spliced into six forms [77], and miR-298 may act upon a sequence within a specific exon. Using RNAhybrid [50, 68], an algorithm that can predict noncanonical [78] miRNA interactions, we found a noncanonical predicted site for miR-298 on the tau 3′-UTR. “Noncanonical” binding has turned out to be remarkably common for miRNA activity [79–83]. However, it is also possible that miR-298 reduction of tau isoforms will be an indirect effect; viz a viz a different intermediate protein. In the future, mutagenizing the putative noncanonical site on the 3′UTR would determine whether miR-298 modulates tau expression directly or indirectly. In addition, miRNA regulation/disruption of mRNA splicing through indirect pathways does exist [84, 85], including in AD [84]. A post hoc query of the DIANA-microT database [45] revealed miR-298 interactions with spliceosome components DDX17, HNRNPL, and RBM74, among others. However, miR-298 may instead alter tau phosphorylation through acting on c-Jun N-terminal kinases (JNK) [74, 86], and JNK alters tau phosphorylation [87]. Hence, we posit that miR-298 modulated the expression of 55 and 50 kDa tau protein moieties either directly via tau isoform transcript regulation, or indirectly via spliceosome or tau kinase regulation. Further mechanistic studies will be required to elucidate the mechanism and functional consequences of this miR-298-mediated tau pathway. In addition, we post hoc queried the DIANA-microT database and found interactions between miR-298 and the MAPK1 and PHKG2 kinases, both of which also phosphorylate tau. Given that a miR-298 SNP associated with differential tau phosphorylation in the SNP association clinical sample, the latter is more likely. However, each possibility would be worth investigating in future studies. Regarding tau, we observed a significant decrease in 55 kDa and 50 kDa tau protein bands and non-significant decrease in 48 kDa and total tau proteins. Protein signals were scanned and quantified independently by different raters. We urge caution in interpretation, since the 50 kDa band, though light and distinct, was not well separated from the much darker and more widely expressed 48kDa band. Attempts to resolve the two by adjusting exposure of ECL signal or lowering loading protein samples would have rendered both the 50kDa and 55kDa bands difficult or impossible to detect. However, if the analysis were done for each individual band’s densitometry as a fraction of total tau signal, we found the same results with 55kDa, but the proportion of 48kDa tau vs. total tau was elevated after miR-298 treatment (data not shown). Repeating the experiment with several mixed brain cultures from HFB samples of similar demography and clinical history could be tried, beyond the three cases we tried (data not shown). Unfortunately, obtaining a supply of HFB remains a critical challenge [88]. Finally, in our experiments we observed 3 major tau forms, and not the six tau isoforms observed generally in case of adult human brain [89]. The progression of tau expression from 3 to 6 forms, with or without 3 repeats, and consequent tau’s expanding role from the fetal to adult stage, would warrant an important area of further investigation.

Potential complexity of miR-298 activity could reach beyond even these pathways, given that miR-298 participates in interspecies communication of some form. That is to say, miR-298 is among the known miRNAs whose levels are altered by the gut microbiome, specifically in the ilium [90]. In addition, microbiome manipulation in the ilium resulted in disruption of several proteins’ levels, including proteins regulated by miR-298. These included the chaperone FK06 binding protein 51 kDa (FKBP5). FKBP5 mediates oligomerization of tau [91]. Another potential miR-298 target disrupted by manipulation of the ilium microbiome was stearoyl-CoA desaturase enzyme 1 (SCD1). Desaturases underlie some tau hyperphosphorylation pathways [92], and SCD1 in particular is elevated in brains of AD patients [93]. How much this may be connected to results in our cell culture studies, of course, remains to be seen.

On searching for reported SNPs within the MIR-298 gene, we found one SNP (rs71828623) with a G/T mutation within the seed sequence of MIR-298. However, it had no reported association with any AD biomarker or subphenotype. We also found other SNPs in proximity of the MIR-298 gene, two of which were associated with the AD biomarkers CSF phosphorylated tau (rs6070629) and CSF Aβ42 (rs79259988). The specific phosphorylation variant pT181-Qß associated with rs6070629 has also been tested as a vaccine antigen. This vaccine induced robust, protective immunity against tauopathy [94].

The predominant model of miRNA protein translation suppression is that RISC binds to mRNA and destabilizes the molecule [95]. We, therefore, tested whether or not induction of overexpression of miR-298 would alter overall levels of mRNA for APP, BACE1, and tau. We found that APP and BACE1 mRNA levels were significantly reduced, while tau reduction was not significant. Given that miR-298 reduced levels of only a single tau variant, this is not surprising, since the activity of miR-298 on tau may be through inducing alternate splicing and/or phosphorylation.

When we examined expression of miR-298 in the human brain by qPCR, we found that miR-298 levels were reduced in AD, but only in the TL (BA 21/22). Both cerebellum and PCC had no differences by disease status. Interestingly, pilot miRNA sequencing of non-AD and AD PCC tissue from the Rush cohort (*n* = 10/group) revealed a ~3-fold upregulation of miR-298 in AD. The discrepancy between these data and the qPCR-based results may be technology-specific, as a systematic comparison of microarray profiling, qPCR, and next-generation sequencing of miRNAs demonstrated that miRNA levels derived from sequencing and qPCR could differ by as much as 5 log2 [96]. Furthermore, we only found the difference when looking at relative (ΔΔCt) measurement. When we looked at absolute mass of miRNA, no apparent AD-related change existed. Conclusive resolution of these apparent paradoxes will require further work. However, we do note reports that indicate both absolute and relative quantification of mRNA may be of use [97]. Technical hurdles, such as circumventing the blood–brain barrier, still exist, but advancing techniques, such as viral, liposomal, or convection-enhanced delivery that may yet overcome this issue [98–100]. Finally, a better picture would be obtained by including brains from donors with subjective cognitive decline or MCI [101, 102], since the consensus of the field has evolved to include the premise that critical changes in brains are most likely to happen well before any overt clinical presentation. Nevertheless, our demonstration that miR-298 modulates APP, BACE1, and tau isoform or phosphorylation levels has implications for its utility as a drug target.

Currently approved treatments for AD consist of cholinesterase inhibitors and a single NMDA antagonist. While ablation of BACE1 expression and/or activity has been shown to reverse AD pathology in AD-transgenic mice [103], clinical trials have been unsuccessful. In fact, the majority of more recent anti-AD drug candidates have been BACE1 inhibitors, but have been withdrawn due to toxicity or lack of efficacy. The possibility may exist that a sufficiently high dose of a BACE1 inhibitor may be inherently toxic, due to BACE1’s multiple other substrates [104–106]. Treatments that target Aβ directly have also not fared well. All these approaches share a single feature: They pick one aspect of AD and target it exclusively. AD is a multifaceted disorder. Even at its simplest, it cannot be reduced to a tauopathy or an amyloidosis; AD is a tauopathy and an amyloidosis. Therapeutics that target tau are being researched for AD, but trials are concentrating on the N terminus of the tau protein, which is often truncated in AD [107]. An alternative is an antibody-based approach [108], which is being tested for progressive supranuclear palsy. However, anti-Aβ treatments for AD appear to be meeting limited success. Perhaps a single target with multivalent effects (e.g., APP, BACE1, and p-tau), may prove more useful. In some cases, a single miRNA may play a role in multiple disorders, such as the potential for miR-101 to be involved in both AD and pulmonary fibrosis [109]. As a methodological note, our choice of hsa-miR-186 as a negative control for APP regulation was not coincidental. This miRNA species is known to downregulate BACE1 and its downregulation during aging may increase risk of AD [108, 111].

This present work significantly advances the field. Specific miRNAs play a critical role in brain aging, neuroinflammation, neurodegeneration, and brain trauma [112] miRNAs play major roles in cognitive impairment in both AD-like pathology and dementia with Lewy bodies [113, 114]. For example, miR-455–3p may have protective effects against abnormal APP processing and Aβ-toxicity in AD [115]. Furthermore, specific miRNAs may associate with mitochondrial metabolism, cholinergic imbalances, and epileptiform activity [116, 117]. Notably, a family of miRNAs that has strong implications in multiple disorders, including neurodegenerative (miR-15/107)  is significantly expressed in human brain tissues [118, 119]. At the functional level, neuronal-expressed microRNA-targeted pseudogenes compete with coding genes in the human brain [120]. How miR-298 could exert other roles beyond what we report here warrants further investigation.

We identified miR-298 as a novel therapeutic target to reduce expression of APP and BACE1 and reduce levels of two moieties of t-tau. In addition, levels of Aβ peptides are significantly reduced, presumably by reduction of both the APP substrate and the rate-limiting BACE1 enzyme. This result has applications to both understanding the mechanism of AD as well as treating the neurodegenerative disorder. Thus, miR-298 has the potential to be a regulator of multiple proteins that are critical in AD etiology and development.

## Supplementary information

Supplementary Material-

## Data Availability

The datasets generated during the current study are available from the corresponding author on reasonable request.
