# Supplementary material for: MicroRNA-298 reduces levels of human amyloid-β precursor protein (APP), β-site APP-converting enzyme 1 (BACE1) and specific tau protein moieties
Source: Mol Psychiatry. 2020 Jan 15;26(10):5636–57. doi: 10.1038/s41380-019-0610-2 (PMC8758483; doi:10.1038/s41380-019-0610-2)
Supplement: Supplementary file 1 — Supplementary Material- [file 41380_2019_610_MOESM1_ESM.pdf]

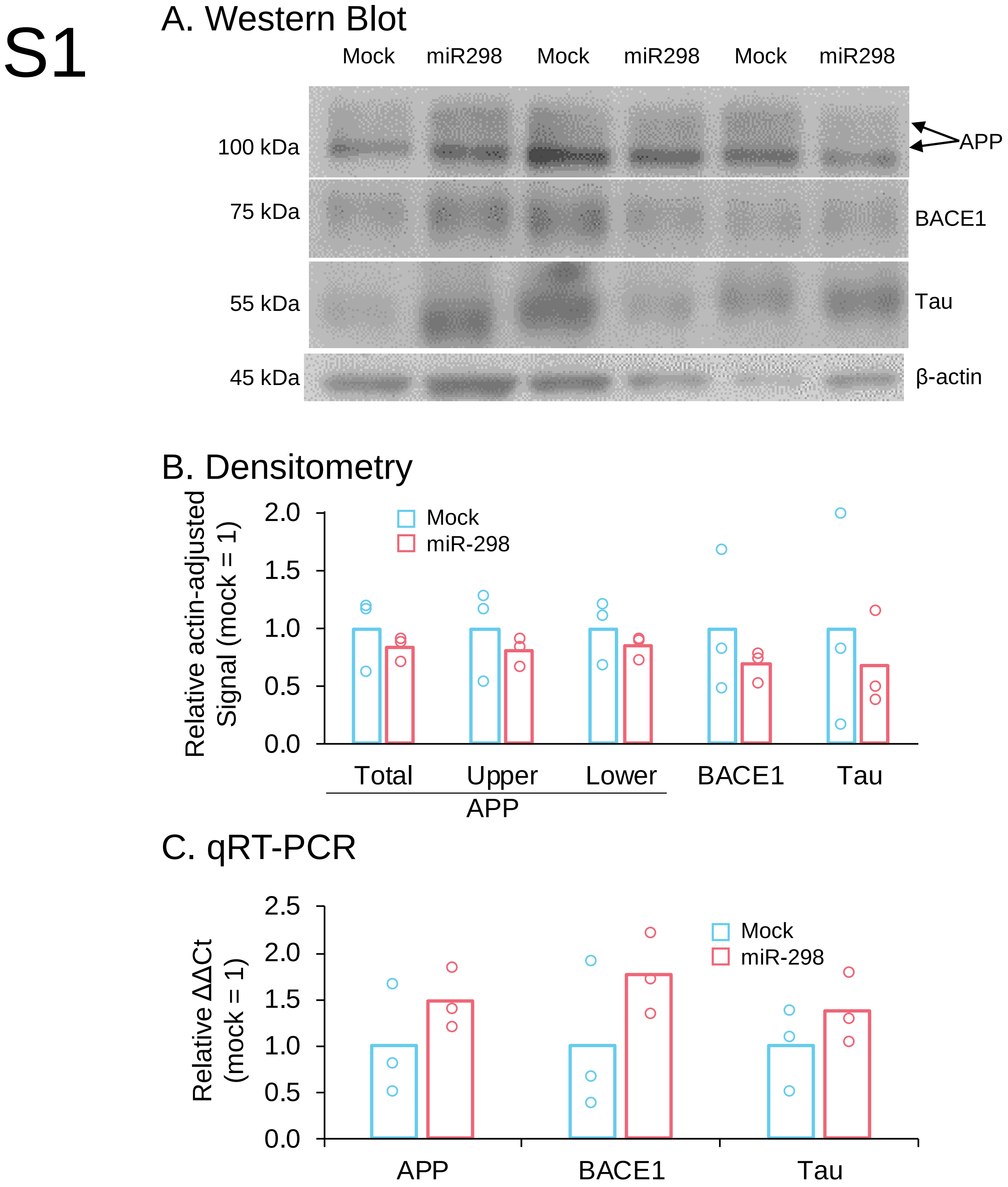

Supplementary Fig. 1. Viral expression of human miR-298 produced non-significant reduction of APP, BACE1, and tau proteins and non-significant elevation of corresponding mRNAs. Mice were infected by tail injection with a viral AAV9 vector that included either mock sequence or human miR-298. Spinal cords were harvested and proteins extracted as described herein. A) Western blotting was performed for APP, BACE, and τ protein. B) Gel was scanned and densitometry adjusted by β-actin signal. No significance was found for miR-298 treatment, although all proteins showed non-significant reduction. Samples from the same animals were also used to produce cDNA for RT-PCR as described herein. miRNA-298 treatment corresponded with elevation of mRNAs for APP, BACE1 and tau, but no changes were significant.
